# Rare presentation of pancreatic schwannoma: a case report

**DOI:** 10.1186/1752-1947-2-268

**Published:** 2008-08-12

**Authors:** Arash Mohammadi Tofigh, Mohammad Hashemi, Behzad Nemati Honar, Fereidoon Solhjoo

**Affiliations:** 1 Department of Surgery, Imam Hussein Hospital, Shahid Beheshti University/MC, Tehran, Iran; 2 Department of Pathology, Imam Hussein Hospital, Shahid Beheshti University/MC, Tehran, Iran

## Abstract

**Introduction:**

Schwannoma is a rare tumor among pancreatic neoplasms. Schwannomas vary in size, and most of them are cystic, mimicking pancreatic cystic lesions. Generally, a definitive diagnosis is made at the time of histological analysis. The mainstay treatment is surgical resection.

**Case presentation:**

We report an unusual presentation of pancreatic schwannoma with abdominal pain and several episodes of cholangitis in a 54-year-old Caucasian (Iranian) man. The condition was not diagnosed pre-operatively and Whipple's procedure was performed.

**Conclusion:**

Pancreatic schwannoma is an important clinical entity to include in the differential diagnosis of pancreatic lesions. Pre-operative diagnosis is difficult but computed tomographic findings may be helpful. The tumor may also have atypical and rare presentations, such as cholangitis and weight loss. For benign tumors, simple enucleation is usually adequate, whereas malignant tumors require standard oncological resection.

## Introduction

Schwannomas are uncommon neoplasms. They are sometimes also referred to as neurilemmomas and usually occur in the extremities, but can also be found in the trunk, head and neck, pelvis, and rectum [1]. Benign types comprise 65% of all neurogenic tumors, but 10%–15% are malignant [2]. Pancreatic schwannomas arise from either sympathetic or parasympathetic fibers and the pancreas is an extremely unusual site of origin for this tumor. These tumors predominantly affect adults, with an equal sex distribution [3]. We report a patient with a pancreatic head tumor presenting with cholangitis, which was found to be a schwannoma, a rare case with an unusual presentation.

## Case presentation

A 54-year-old Caucasian (Iranian) man presented at our hospital with a history of intermittent epigastric pain and several episodes of cholangitis over the previous year. The pain ranged from moderate to severe and was associated with weight loss, nausea, vomiting, and intermittent jaundice. On physical examination, he was febrile (oral temperature, 38°C) and mildly icteric. His abdomen was soft, non-distended, with no palpable mass, but mildly tender to palpation in the epigastrium.

On laboratory tests, he showed mild direct bilirubinemia (total: 4 mg/dl; direct: 2.5 mg/dl), but normal hemoglobin, liver function, and amylase. The tumor markers carbohydrate antigen (CA) 19-9 and carcinoembryonic antigen (CEA) were in the normal ranges.

A dynamic computed tomographic (CT) scan demonstrated a 30 × 2.5 mm mass in the pancreatic head area. Upper gastrointestinal (GI) side-view endoscopy showed a normal papilla of Vater. Based on the patient's history, clinical findings, and CT images, he was taken to the operating theater for the Whipple procedure.

**Figure 1:**
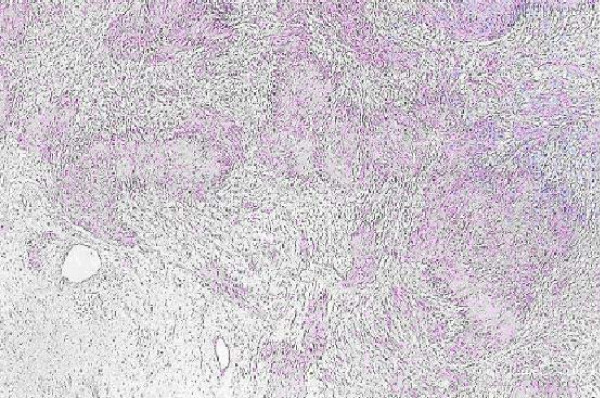
Pathological analysis showed a schwannoma consisting of spindle cells arranged in a palisading fashion.

With laparotomy, a mass was found in the head of the pancreas, without encasement of the superior mesenteric artery or portal vein. No mesenteric lymphadenopathy, peritoneal implants, or liver lesions were found. A classic pancreatoduodenectomy was performed. The pathological analysis showed a schwannoma consisting of spindle cells arranged in a palisading fashion (Fig. 1). Immunohistochemical staining showed that the tumor cells were diffusely and strongly positive for S100 protein.

Ten months after the operation, the patient was completely symptom free.

## Discussion

Less than 26 cases of pancreatic schwannoma have been reported in the literature. They vary in size from 1.5 cm to 20 cm in diameter. The majority of these tumors were found in the head and body of the pancreas. Generally, they were slow growing and originated from the peripheral epineurium of either autonomic sympathetic or parasympathetic fibers [3]. More than half of them were cystic and benign [1,4].

Nonspecific abdominal pain was the most commonly reported symptom but weight loss, jaundice, and GI bleeding were also reported [4]. There is no report of recurrent cholangitis (observed in our patient) as a presenting symptom of the tumor. A CT scan is often the initial study of choice. CT findings usually show well-defined and hypodense tumors, with encapsulation and cystic degeneration [5].

**Figure 2:**
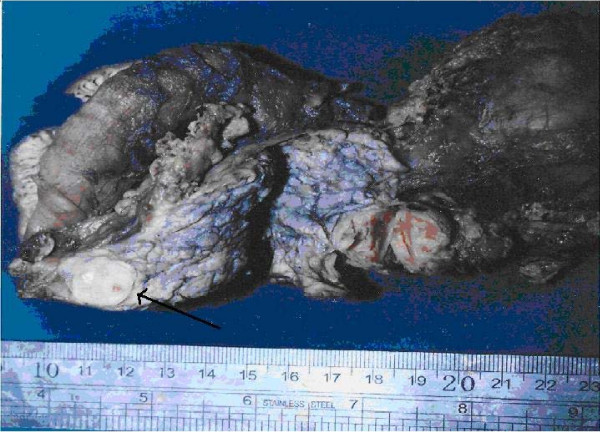
Resected specimen showing the gross appearance of the tumor (arrow).

The diagnosis of pancreatic schwannoma may be complicated by an inadequate amount of specimen or by deficiencies in the specimen-collecting techniques used. Fine-needle aspiration correctly diagnoses only one of eight histologically proven schwannomas [6]. Pancreatic schwannoma is usually diagnosed after histopathological analysis [7,8]. In our patient, an encapsulated homogeneous tan-yellow round nodule was found, 3 cm in diameter, situated on the superior anterior aspect of the pancreatic head (Fig. 2). Because the malignant transformation of pancreatic schwannomas is uncommon, simple enucleation is usually sufficient if the pathology is confirmed before surgery. Otherwise, oncological resection (Whipple procedure) is indicated [8]. To date, no documented recurrent case has been reported after either mode of resection. In this patient, the tumor was resectable and the Whipple procedure was performed.

## Conclusion

Pancreatic schwannoma is an important clinical entity to include in the differential diagnosis of pancreatic lesions. Pre-operative diagnosis is difficult but CT findings may be helpful. The tumor may also have atypical and rare presentations, such as cholangitis and weight loss. For benign tumors, simple enucleation is usually adequate, whereas malignant tumors require standard oncological resection.

## Consent

Written informed consent was obtained from the patient for the publication of this case report and any accompanying images. A copy of the written consent is available for review by the Editor-in-Chief of this journal.

## Competing interests

The authors declare that they have no competing interests.

## Authors' contributions

AMT, MH, BNH, and FS were all involved in the management of the patient, and in writing the case report. All authors have read and approved the manuscript.
